# Structural analysis of β-glucosidase mutants derived from a hyperthermophilic tetrameric structure

**DOI:** 10.1107/S1399004713032276

**Published:** 2014-02-27

**Authors:** Makoto Nakabayashi, Misumi Kataoka, Yumiko Mishima, Yuka Maeno, Kazuhiko Ishikawa

**Affiliations:** a Biomass Refinery Research Center, National Institute of Advanced Industrial Science, 3-11-32, Kagamiyama, Higashi-Hiroshima, Hiroshima 739-0046, Japan

**Keywords:** protein engineering, crystal engineering, intermolecular interactions, thermostable enzymes, biomass

## Abstract

β-Glucosidase from *Pyrococcus furiosus* (BGLPf) is a hyperthermophilic tetrameric enzyme which can degrade cellooligosaccharides to glucose under hyperthermophilic conditions and thus holds promise for the saccharification of lignocellulosic biomass at high temperature. Prior to the production of large amounts of this enzyme, detailed information regarding the oligomeric structure of the enzyme is required. Several crystals of BGLPf have been prepared over the past ten years, but its crystal structure had not been solved until recently. In 2011, the first crystal structure of BGLPf was solved and a model was constructed at somewhat low resolution (2.35 Å). In order to obtain more detailed structural data on BGLPf, the relationship between its tetrameric structure and the quality of the crystal was re-examined. A dimeric form of BGLPf was constructed and its crystal structure was solved at a resolution of 1.70 Å using protein-engineering methods. Furthermore, using the high-resolution crystal structural data for the dimeric form, a monomeric form of BGLPf was constructed which retained the intrinsic activity of the tetrameric form. The thermostability of BGLPf is affected by its oligomeric structure. Here, the biophysical and biochemical properties of engineered dimeric and monomeric BGLPfs are reported, which are promising prototype models to apply to the saccharification reaction. Furthermore, details regarding the oligomeric structures of BGLPf and the reasons why the mutations yielded improved crystal structures are discussed.

## Abstract 2

Substitutive mutations that convert a tetrameric β-glucosidase into a dimeric state lead to improvement of its crystal quality.

## 1. Introduction   

Most of the biomass on earth is composed of cellulosic materials, which can be converted into biofuels or bio-based materials (Bayer & Lamed, 1992 ▶; Farrell *et al.*, 2006 ▶; Joshi & Mansfield, 2007 ▶; Ragauskas *et al.*, 2006 ▶). The production of biofuels or bio-based materials from cellulosic biomass requires saccharification to obtain fermentable sugars. In nature, cellulolytic microbes typically produce three categories of cellulases which catalyze the conversion of cellulose into glucose: endoglucanases (EGs), cellobiohydrolases (CBHs) and β-glucosidases (BGLs) (Baldrian & Valásková, 2008 ▶; Stricker *et al.*, 2008 ▶; Tomme *et al.*, 1995 ▶). Cellulase systems using these three types of enzymes show potential for the complete enzymatic saccharification of cellulose on an industrial scale. To date, *Trichoderma reesei* has been considered to be a strong cellulolytic and xylanolytic candidate microorganism. However, complete saccharification of cellulose is not accomplished by the cellulases from *T. reesei* because of its low BGL activity (Fang *et al.*, 2009 ▶). To overcome this problem, a BGL exhibiting high activity is required. At the same time, much effort has been made to find a thermophilic cellulase system for the industrial conversion of biomass because the enzymatic degradation of biomass at high temperature would provide advantages such as limiting bacterial contamination and increasing the reactivity and substrate solubility.

Recently, an endocellulase (EGPh; family 5) from the hyperthermophilic archaeon *Pyrococcus horikoshii* was identified and recombinant EGPh was expressed in *Escherichia coli* (Ando *et al.*, 2002 ▶; Kashima *et al.*, 2005 ▶; Kim *et al.*, 2007 ▶, 2008 ▶). EGPh exhibits progressive hydrolytic activity, releasing cellobiose after an initial endo-type attack on cellulose. Hyperthermophilic archaeal BGL (BGLPf; family 1) has also been isolated from *P. furiosus* (Bauer *et al.*, 1996 ▶; Kaper *et al.*, 2000 ▶) and holds significant potential for the complete saccharification of cellulose at high temperature when combined with EGPh (Kim & Ishikawa, 2010 ▶). BGLPf exhibits high hydrolytic activity towards cellooligosaccharides at high temperatures (Kaper *et al.*, 2000 ▶). Furthermore, the tetrameric structure of BGLPf has been solved at the somewhat low resolution of 2.35 Å (Kado *et al.*, 2011 ▶). However, some issues need to be addressed before BGLPf can be used industrially. One is that BGLPf forms a tetrameric structure. Industrial use will require large amounts of BGLPf, but oligomeric enzymes are difficult to be secreted in large quantities. To address this issue and to improve the resolution of the crystal structural analysis, we created dimeric and monomeric mutants that retain the intrinsic activity of the tetrameric enzyme. Here, we present the biophysical and biochemical properties of dimeric and monomeric mutant BGLPfs and discuss the crystal structure of the oligomeric BGLPf.

## 2. Materials and methods   

Unless otherwise noted, all experiments were performed at room temperature.

### 2.1. Protein expression and purification   

To obtain the mutant BGLPf genes, we used the QuikChange Site-Directed Mutagenesis Kit (Stratagene) and performed PCR with the pET-11d/BGLPf plasmid as a template. Mutagenesis experiments to obtain BGLPf-M3 were divided into three discrete PCR experiments, by which R170A, R220A and Y227F mutations were introduced. Subsequent mutagenesis experiments to obtain BGLPf-M4a, BGLPf-M4b and BGLPf-M4c were performed using the pET-11d/BGLPf-M3 plasmid as a template.

Recombinant wild-type BGLPf (BGLPf-WT) was expressed and purified using the method described previously (Kado *et al.*, 2011 ▶). Recombinant BGLPf and its mutants were expressed in *Escherichia coli* BL21 (DE3) cells (Novagen). Cell cultures were grown at 37°C in Luria Broth medium containing 100 mg ml^−1^ ampicillin sodium salt until the optical density at 600 nm (OD_600_) reached 0.3. Cell cultures were subsequently grown at 16°C until the OD_600_ reached 0.6 and were then induced for 6 h with 1 m*M* isopropyl β-d-1-thiogalactopyranoside (IPTG) at 16°C.

The harvested cells were lysed on ice by sonication in 50 m*M* Tris–HCl pH 8.0 with 2 m*M* phenylmethylsulfonyl fluoride. The cell lysate was centrifuged at 9000*g* for 30 min at 4°C. The supernatant was fractionated with ammonium sulfate to 80% saturation. After centrifugation, the pellet was resuspended in 50 m*M* Tris–HCl pH 8.0 and then dialyzed overnight at 4°C against 50 m*M* Tris–HCl pH 8.0. The lysate was loaded onto a HiTrap Q anion-exchange column (GE Healthcare Bio­sciences) equilibrated with 50 m*M* Tris–HCl pH 8.0 and eluted with a linear gradient of 0–1.0 *M* NaCl. The eluted solution was fractionated with ammonium sulfate to 40% saturation. After centrifugation, the supernatant was loaded onto a hydrophobic HiTrap Phenyl column (GE Healthcare Bio­sciences) equilibrated with 20 m*M* Tris–HCl buffer pH 8.0 containing 1.5 *M* ammonium sulfate and eluted with a linear gradient of 1.5–0 *M* ammonium sulfate. The eluted solution was loaded onto a HiLoad 26/60 Superdex 200 pg column (GE Healthcare Biosciences) with 20 m*M* Tris–HCl buffer pH 8.0 containing 0.1 *M* NaCl.

Recombinant BGLPf-M4a, BGLPf-M4b and BGLPf-M4c were expressed and purified using a similar method as for BGLPf-M3, but not including the ammonium sulfate fractionation and HiTrap Phenyl chromatography steps. The purity and size of the proteins were assessed by reducing SDS–PAGE.

The protein concentration was determined from the UV absorbance at 280 nm using molar extinction coefficients as calculated from the protein sequences using a standard method (Gill & von Hippel, 1989 ▶).

### 2.2. Crystallization   

Purified BGLPf-M3 solution was concentrated to about 8.0 mg ml^−1^ by ultrafiltration. Crystals were obtained using the hanging-drop vapour-diffusion method using a series of precipitant solutions consisting of 0.1 *M* HEPES–NaOH pH 7.0–7.8, 1.0–1.6 *M* ammonium dihydrogen phosphate. Crystallization droplets were prepared by mixing 2.4 µl protein solution and 1.2 µl precipitant solution, and the droplets were equilibrated against 500 µl precipitant solution at 20°C. It took 1–3 d to obtain crystals of suitable quality for X-ray diffraction.

### 2.3. Diffraction experiments and structure analysis   

Prior to diffraction data collection, crystals were soaked in cryoprotectant solution consisting of the precipitant solution containing 10 or 20%(*v*/*v*) glycerol. The crystals were first soaked in solution with 10% glycerol and then in solution with 20% glycerol. Diffraction data sets were collected at −173°C in a stream of nitrogen gas on beamline BL44XU at SPring-8, Hyogo, Japan. Reflections were recorded using an oscillation range of 0.5° per image. Diffraction data were indexed, integrated and scaled using *HKL*-2000 (Otwinowski & Minor, 1997 ▶). The structure of BGLPf-M3 was solved by molecular replacement with *MOLREP* (Vagin & Teplyakov, 2010 ▶) and finalized sets of atomic coordinates were obtained after iterative rounds of model modification with *Coot *(Emsley & Cowtan, 2004 ▶) and refinement with *REFMAC*5 (Murshudov *et al.*, 2011 ▶) and *CNS* (Brünger *et al.*, 1998 ▶) using rigid-body refinement, positional minimization, water-molecule identification and individual isotropic *B*-value refinement.

Superimposition between the structure models was carried out using *ProFit*. Pictures of the BGLPfs were drawn using *PyMOL* (http://www.pymol.org).

### 2.4. Evaluation of molecular sizes   

The oligomeric states of BGLPf-WT and the mutants were examined by gel filtration using a HiLoad 26/60 Superdex 200 pg column and the dynamic light-scattering (DLS) method (using an instrument custom-built by Associate Professor Shinpei Tanaka, Hiroshima University, Hiroshima, Japan).

The samples of the mutants and the wild type for gel filtration were in 50 m*M* Tris–HCl buffer pH 8.0 containing 0.15 *M* NaCl. The flow rate was adjusted to 2.0 ml min^−1^ and the time course of the absorbance at 280 nm was monitored. The samples of the mutants and the wild type were adjusted to a concentration of 10 mg ml^−1^ in 20 m*M* Tris–HCl buffer pH 8.0 for DLS. DLS measurements were performed at 20°C.

### 2.5. Evaluation of thermostabilities   

Differential scanning calorimetry (DSC) measurements were carried out using a Nano DSC II instrument (TA Instruments, Delaware, USA) with platinum tubing cells with a volume of 0.3 ml by Associate Professor Harumi Fukada, Osaka Prefecture University, Osaka, Japan. Prior to the DSC experiment, the samples of the mutants and the wild type were dialyzed against 50 m*M* sodium phosphate buffer pH 7.0 and adjusted to a concentration of 10 mg ml^−1^. The experiments were performed over the temperature range 35–125°C at a scan rate of 1°C min^−1^.

Prior to the measurement of the residual activities of the mutants and the wild type, the purified enzymes were incubated for 10 min in 50 m*M* Tris–HCl buffer pH 7.2 at the following temperatures: 50, 60, 65, 70, 75, 80, 85 and 90°C. After the heat treatment, the residual activity of each mutant or the wild type was assayed under standard conditions containing the enzyme at 0.075 mg ml^−1^ for 10 min at 40°C using 10 m*M* cellobiose as the substrate. The residual activity was expressed as the concentration of glucose produced (%).

## 3. Results   

### 3.1. Revisiting the tetrameric structure of BGLPf   

**Figure 1:**
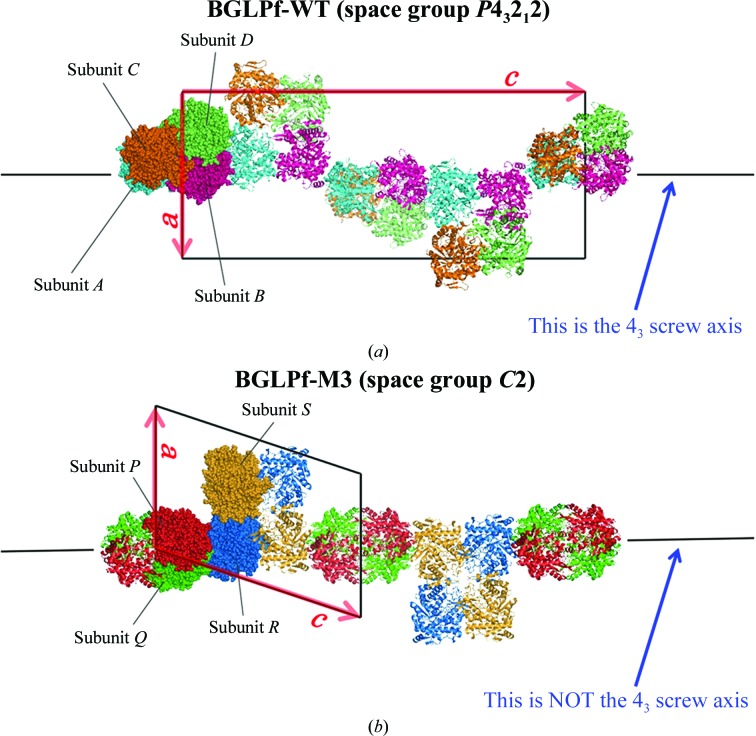
Crystal structures of (*a*) BGLPf-WT and (*b*) BGLPf-M3. (*a*) One tetramer with the wild-type sequence occupies the asymmetric unit and is shown as a space-filling model in cyan (subunit *A*), pink (subunit *B*), orange (subunit *C*) and lime (subunit *D*). The other molecules related by crystallographic symmetry are shown as ribbon models. The unit cell of BGLPf-WT is shown as a black rectangle and the lattice vectors *a* and *c* are shown as red arrows. The 4_3_ screw axis parallel to the lattice vector *c* is shown as a pair of black lines. (*b*) Two dimers (*PQ* and *RS*) of BGLPf-M3 occupy the asymmetric unit and are shown as space-filling models in red (subunit *P*), green (subunit *Q*), blue (subunit *R*) and yellow (subunit *S*). The other molecules related by crystallographic symmetry are shown as ribbon models. The unit cell of M3 is shown as a black parallelogram and the lattice vectors *a* and *c* are shown as red arrows. The arrangement of BGLPf-M3 is similar to that of BGLPf-WT; however, no 4_3_ screw axis is observed in the crystal because the *RS* dimer is not identical to the *PQ* dimer.

The hyperthermophilic β-glucosidase from *P. furiosus* (BGLPf) was first crystallized by Kaper *et al.* (2000 ▶). They reported diffraction data acquired at 3.3 Å resolution, but no structural model of BGLPf was presented (Kaper *et al.*, 2000 ▶). The structure of BGLPf was first solved and modelled by Kado *et al.* (2011 ▶) (Fig. 1 ▶ *a*) through iterative improvement of the methodology for crystallization and data collection. The structure was determined at 2.35 Å resolution and showed a stable homotetrameric structure. Because the structure was solved at low resolution, we attempted to obtain good crystals of BGLPf using several crystallization screening methods. We revisited the BGLPf structure using protein-engineering methods by designing dimeric or monomeric BGLPf mutants. The tetrameric BGLPf without mutations is referred to as BGLPf-WT (see Supplementary Table S1^1^). As described previously (Kado *et al.*, 2011 ▶), the crystal of BGLPf-WT belonged to space group *P*4_3_2_1_2 and its asymmetric unit comprises homotetrameric molecules consisting of four subunits named subunits *A*, *B*, *C* and *D* (PDB entry 3apg; Fig. 1 ▶ *a*), which associate to form 222 point-group symmetry (Kado *et al.*, 2011 ▶). Detailed examination of the interface between the tetramer subunits using *PISA* (*Protein Interfaces, Surfaces and Assemblies*; Krissinel & Henrick, 2007 ▶) allowed us to determine whether or not a protein adopts a polymeric state on the basis of the crystal structure. *PISA* showed that BGLPf-WT should form a tetrameric assembly and that several residues are involved in subunit contacts. The structure of BGLPf-WT exhibits two discrete types of subunit interactions. One type is between subunits *A* and *B* (see Supplementary Table S2 and Supplementary Figs. S1 and S2) and is almost identical to the interaction between subunits *C* and *D*. The other is between subunits *A* and *C* (Table 1 ▶ *a*) and is almost identical to that between subunits *B* and *D*; it is distinctly different from the *A*–*B* (*C*–*D*) interaction. Our previous study indicated that the BGLPf-WT tetrameric structure is stable and that the dimer from the tetrameric structure does not easily dissociate into individual monomers even when treated with extreme conditions such as heating with SDS and a reducing agent (Kado *et al.*, 2011 ▶). Structural analysis of the tetramer indicated that the interaction between *A* and *B* (or *C* and *D*) (Supplementary Fig. S1) contributes to maintaining the stable tetrameric structure.

### 3.2. Substitutive mutations for disrupting the *A*–*B* interactions   

Structural analysis of the interface between subunits *A* and *B* (and *C* and *D*) (Supplementary Fig. S1) with *PISA* identified Arg170, Arg220 and Tyr227 as the key residues (Supplementary Fig. S2). Therefore, in order to disrupt these intersubunit interactions, we constructed a BGLPf mutant (R170A/R220A/Y227F) in which Arg170, Arg220 and Tyr227 were substituted by Ala, Ala and Phe, respectively. This three-point mutation of BGLPf is referred to as BGLPf-M3 (see Supplementary Table S1). Purification of BGLPf-M3 required elimination of the typical heat-treatment step (85°C, 30 min) owing to the instability of the mutant; it was replaced by ammonium sulfate fractionation, anion-exchange chromatography (HiTrap Q), hydrophobic chromatography (HiTrap Phenyl) and gel filtration (Superdex 200). The purity and homogeneity of the protein was assessed by SDS–PAGE. BGLPf-M3 migrated with an apparent relative molecular mass of about 55 kDa (data not shown) after heating at 95°C in reducing SDS–PAGE loading buffer. In order to obtain more detailed structural information on BGLPf, we attempted to prepare a crystal of BGLPf-M3. Purified BGLPf-M3 could not be crystallized using the same conditions for as used for BGLPf-WT. BGLPf-M3 was crystallized using the hanging-drop vapour-diffusion method in precipitant solution consisting of 0.1 *M* HEPES–NaOH pH 7.0–7.8, 1.0–1.6 *M* ammonium dihydrogen phosphate.

Crystal structural analysis showed that the BGLPf-M3 crystals belonged to space group *C*2, which is different from that of BGLPf-WT (Fig. 1 ▶). Diffraction data were improved to 1.70 Å resolution and the structure was refined (Table 2 ▶). These are the highest resolution data reported to date for any BGLPf structure (Kaper *et al.*, 2000 ▶; Kado *et al.*, 2011 ▶). Four subunit molecules per asymmetric unit gave a crystal volume per protein mass (Matthews, 1968 ▶; *V*_M_) of 3.94 Å^3^ Da^−1^ and a solvent content of 68.8%(*v*/*v*), which are similar to those for BGLPf-WT. The four monomers of BGLPf-M3 in the asymmetric unit were named subunits *P*, *Q*, *R* and *S* (Fig. 1 ▶). The root-mean-square (r.m.s.) deviations of the C^α^-atom positions (1–471) among subunits *P*, *Q*, *R* and *S* are less than 0.27 Å. Furthermore, the r.m.s. deviations of the C^α^-atom positions (1–471) of subunits *P*, *Q*, *R* and *S* of BGLPf-M3 compared with subunit *A* of BGLPf-WT are between 0.27 and 0.31 Å. These results indicate that the overall structure of the monomer is not influenced by the mutations. Molecules of glycerol, which was used as a cryoprotectant, were observed in the putative active site of each of the four monomers of BGLPf-M3 and were also observed in the BGLPf-WT crystal structure.

The determination of accurate coordinates was aided by the improved resolution. The electron-density map of BGLPf-M3 revealed *cis*-type peptide bonds at two sites in each subunit: between Pro224 and Pro225 and between Trp410 and Ser411 (Supplementary Fig. S3). These *cis* bonds were also implied in the BGLPf-WT structure.

**Figure 2:**
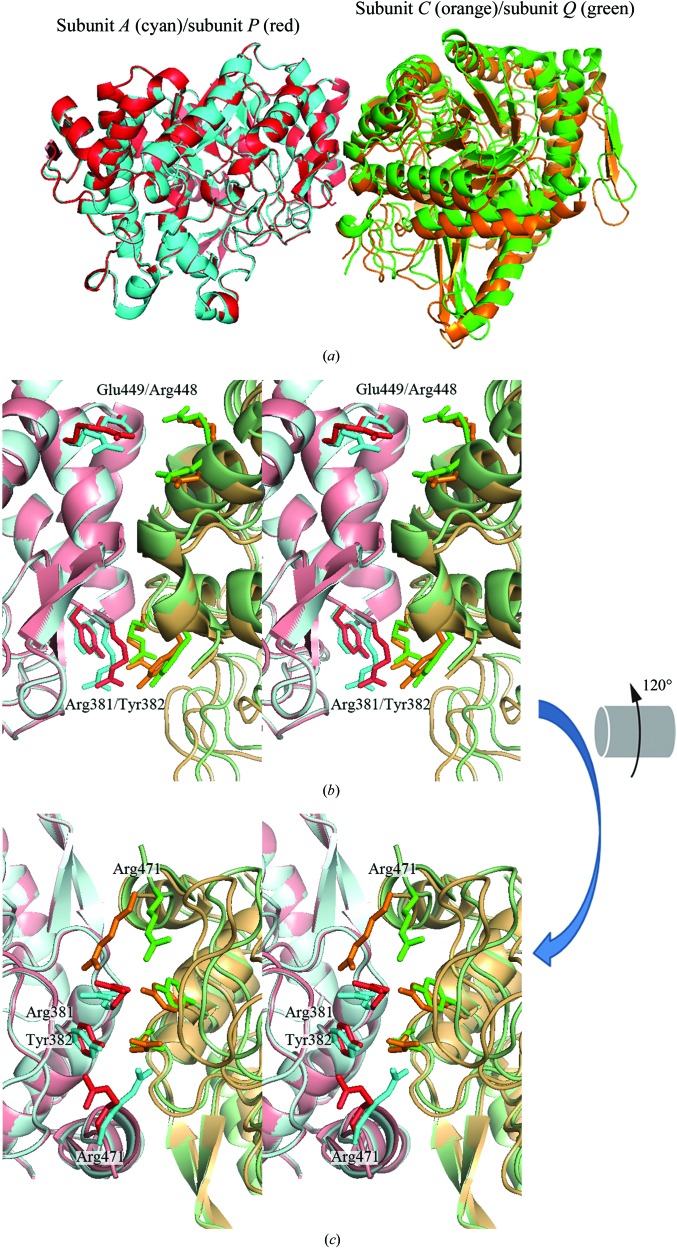
Comparing the *PQ* dimer with the *AC* dimer derived from BGLPf-WT. Sub­units *A* and *C* derived from BGLPf-WT are shown in cyan and orange, respectively, and subunits *P* and *Q* derived from BGLPf-M3 are shown in red and green, respectively. The *PQ* dimer is superimposed on the *AC* dimer. The *P*–­*Q* dimeric interface is similar to the *A*–*C* interface. The Arg381 and Tyr382 side chains contribute to both the *P*–*Q* and *A*–*C* interactions, whereas the Arg448 and Glu449 side chains do not contribute to the *P*–*Q* or *A*–*C* inter­actions (*b*) (stereo diagram). The side chain of Arg471 only contributes to the *A*–*C* interaction (*c*) (stereo diagram) and corresponds to interactions AC-5 and AC-6 in Table 2 ▶(*a*). This side-chain interaction of Arg471 is a remarkable difference between the *PQ* and *AC* dimers. (*b*) is in the same orientation as (*a*). (*b*) was rotated 120° around the horizontal axis to generate (*c*).

**Figure 3:**
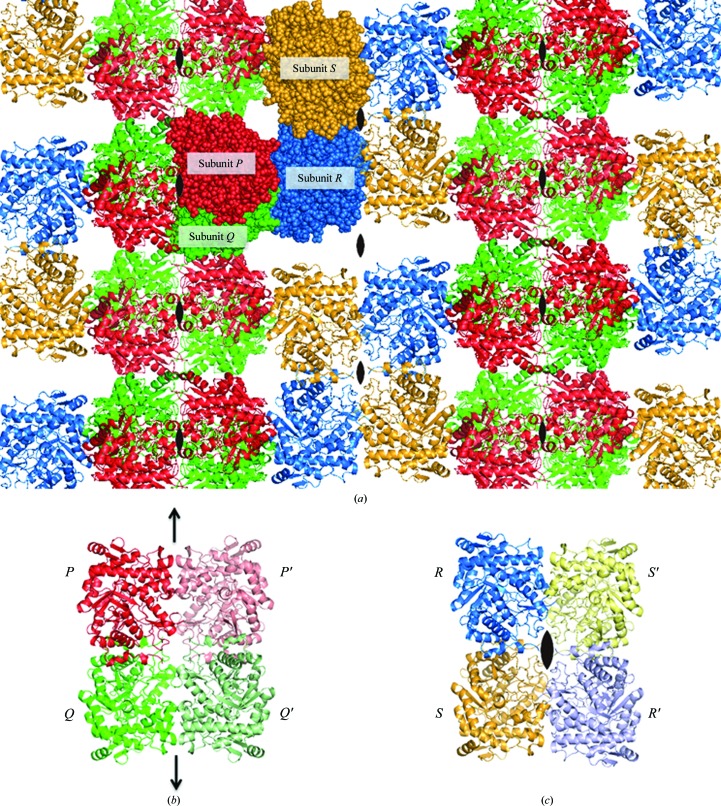
Crystal structure of BGLPf-M3. The four subunits (*P*, red; *Q*, green; *R*, blue; *S*, yellow) are shown as space-filling models in the asymmetric unit. The other molecules related by crystallographic symmetry are shown as ribbon models (*a*). The crystal of BGLPf-M3, which belongs to space group *C*2, has only one unique axis: the axis *b* perpendicular to the page. The positions of the twofold axes are shown by black arrows and convex lenses. (*b*) A dimer, *PQ*, and another dimer, *P*′*Q*′ (related to dimer *PQ* by crystallographic symmetry), form a pseudo-tetramer in the crystal. (*c*) A dimer, *RS*, and another dimer, *R*′*S*′, that is related to dimer *RS* by crystallographic symmetry also form a pseudo-tetramer.

The crystal structure of BGLPf-M3 showed that the dimeric interactions between subunits *P* and *Q* are similar to the *A*–*C* (and *B*–*D*) interactions in BGLPf-WT (Fig. 2 ▶). In contrast, the *R*–*S* interactions are not identical to the *A*–*C* (and *B*–*D*) or *P*–*Q* interactions. Crystallographic packing (Fig. 3 ▶ *a*) shows that BGLPf-M3 can form two distinct types of pseudo-tetrameric structure: a *PP*′*QQ*′ tetramer and an *RR*′*SS*′ tetramer. The *PP*′*QQ*′ tetramer includes a *PQ* dimer and a *P*′*Q*′ dimer that is related to the *PQ* dimer by crystallographic symmetry; likewise, the *RR*′*SS*′ tetramer includes an *RS* dimer and an *R*′*S*′ dimer that is related to the *RS* dimer by crystallo­graphic symmetry (Figs. 3 ▶ *b* and 3 ▶ *c*). The tetrameric interactions of the *PP*′*QQ*′ and *RR*′*SS*′ tetramers are different from those of the *ABCD* tetramer derived from BGLPf-WT. The *PP*′*QQ*′ tetramer structure is maintained by several hydrogen bonds involving the Glu39 side chain, the Ala42 main chain, the Leu228 main chain, the Ala220 main chain and several water molecules. On the other hand, the *RR*′*SS*′ tetramer structure is tethered by several hydrophobic and water-molecule-mediated interactions.

We have no structural data for the three single mutants. However, the pseudo-tetrameric structure of BGLPf-M3 suggests that the three-point mutation is necessary to disrupt the tetrameric structure of BGLPf-WT. Experiments for each of the three single mutants were carried out. However, there was no significant difference in the molecular size and activity among the three mutants and BGLPf-WT (data not shown).

### 3.3. Differences between the *P*–*Q* and *R*–*S* interactions   

**Figure 4:**
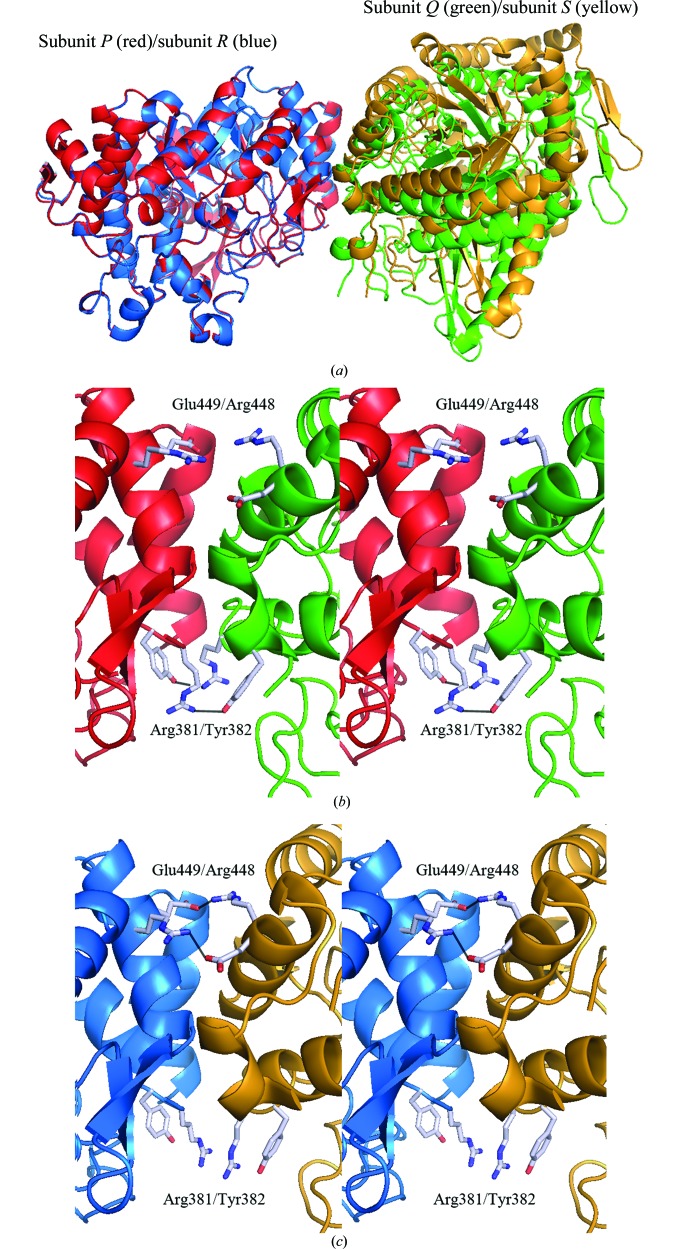
Comparing the *RS* and *PQ* dimers in BGLPf-M3. Subunits *P*, *Q*, *R* and *S* are shown in red, green, blue and yellow, respectively. There is a difference between the *PQ* and *RS* dimers, even though the monomeric structures of *P*, *Q*, *R* and *S* are almost identical. (*a*) When subunit *R* was superimposed on subunit *P*, parts of subunit *S* could not fit onto subunit *Q*. Arg381 and Tyr382 interact, whereas Arg448 and Glu449 displace each other in the *PQ* dimer (*b*). Similarly, Arg448 and Glu449 interacted whereas Arg381 and Tyr382 displaced each other in the *RS* dimer (*c*). Putative interactions are shown by black solid lines.

The crystals show that both BGLPf-M3 and BGLPf-WT have tetrameric structures. However, the tetramer form of BGLPf-M3 is different from that of BGLPf-WT. In individual monomers of BGLPf-M3 subunits *P*, *Q*, *R* and *S* exhibit almost identical structures, but remarkable differences are observed in the dimeric structures between the *PQ* and the *RS* dimers. Superimposing the *RS* dimer on the *PQ* dimer, for instance, shows that it is impossible to fit all the main-chain positions of both dimers (Fig. 4 ▶ *a*), indicating that the structure of the dimeric interface *P*–*Q* is different from the *R*–*S* interface (Figs. 3 ▶ *b* and 3 ▶ *c*). In the BGLPf-WT structure, Arg381 and Tyr382 of subunit *A* interact with Tyr382 and Arg381 of subunit *C*, respectively. Likewise, Arg381 and Tyr382 of subunit *C* also interact with Tyr382 and Arg381 of subunit *A* (Fig. 2 ▶ *b* and Table 1 ▶ *a*). Moreover, Arg471 adjacent to the C-­terminus of each subunit takes part in the *A*–*C* interaction (Fig. 2 ▶ *c* and Table 1 ▶ *a*). In the case of the *PQ* dimer in BGLPf-M3, Arg381 and Tyr382 of subunit *P* interact with Tyr382 and Arg381 of subunit *Q*, respectively, and Arg381 and Tyr382 of subunit *Q* also interact with Tyr382 and Arg381 of subunit *P* (Figs. 2 ▶ *b* and 4*b*). However, Arg471 of subunit *P* or *Q* does not take part in the *P*–*Q* interaction (Fig. 2 ▶ *c* and Table 1 ▶ *b*). In contrast to the *PQ* dimer, Arg381 and Tyr382 in the *RS* dimer do not take part in the interface interactions because the distance between the guanidium group of the Arg381 side chain and the hydroxyl group of the Tyr382 side chain is greater than 6.5 Å (Fig. 4 ▶ *c*). On the other hand, Arg448 and Glu449, which are positioned adjacent in the *RS* dimer, take part alternately in the interaction (Fig. 4 ▶ *c* and Table 1 ▶ *c*); such inter­actions are not observed in the *PQ* dimer nor in the *AC* (or *BD*) dimer. Thus, the *RS* interaction is apparently distinct from the *PQ* interaction in BGLPf-M3 and the *AC* interaction in BGLPf-WT.

### 3.4. Substitutive mutations for disrupting the *R*–*S* and *P*–*Q* interactions   

The crystal structure of BGLPf-M3 exhibits higher resolution than that of BGLPf-WT and also shows several noteworthy interactions between sub­units *R* and *S*. As mentioned above, the *PQ* and *RS* dimers are distinct from each other, even though the *PQ* dimer of BGLPf-M3 is similar to the *AC* dimer of BGLPf-WT. Interactions between Arg381 and Tyr382 were observed in the dimer interface in the *PQ* dimer (Fig. 4 ▶ *b*, Table 1 ▶ *b*), and interactions between Arg448 and Glu449 were observed in the dimer interface in the *RS* dimer (Fig. 4 ▶ *c*, Table 1 ▶ *c*). Furthermore, the main chain of Glu459 contributes to dimer formation in both the *PQ* and *RS* dimers (Table 1 ▶). Two types of alternative dimers (*PQ* and *RS*) are formed in BGLPf-M3, with residues Arg381, Tyr382, Arg448, Glu449 and Glu459 controlling dimer formation (Table 1 ▶). Based on the interactions between Arg448 and Glu449 and between Leu440 and Glu459 observed here, we constructed a series of mutants derived from BGLPf-M3 to create a monomeric form. Individual mutations (R448E, E449R and E459G) were introduced into BGLPf-M3. The E459G mutation was aimed at increasing the flexibility of the main chain adjacent to the dimeric interface. We refer to BGLPfs with these four point mutations as BGLPf-M4a, BGLPf-M4b and BGLPf-M4c (see Supplementary Table S1). These mutants were expressed in *E. coli* BL21 (DE3) and purified using a modified procedure that did not involve heat treatment.

### 3.5. Molecular sizes of the mutants   

The oligomeric states of BGLPf-M4a, BGLPf-M4b and BGLPf-M4c were determined using gel-filtration analysis. Standard protein markers (ferritin, 440 kDa; aldolase, 158 kDa; conalbumin, 75 kDa; ovalbumin, 44 kDa) were used. Each mutant was analyzed individually. The results indicate that BGLPf-M4a (71 kDa) and BGLPf-M4c (50 kDa) are monomeric and that BGLPf-M4b (96 kDa) is not. The dimeric state of BGLPf-M3 (136 kDa) and the tetrameric state of BGLPf-WT (236 kDa) were also confirmed, although the chromatogram of BGLPf-M3 exhibited a small satellite peak corresponding to a tetramer (Supplementary Fig. S4 and Table 3 ▶).

We next employed a dynamic light-scattering (DLS) method to evaluate the sizes of the mutants. The hydrodynamic radii of BGLPf-M4a, BGLPf-M4b, BGLPf-M4c and BGLPf-WT were estimated to be 4.0 ± 1.7, 4.1 ± 1.6, 3.2 ± 1.0 and 5.7 ± 1.1 nm, respectively (Supplementary Figs. S5*a*, S5*c*, S5*d* and S5*e*). These results, except for those for BGLPf-M4b, are consistent with the results obtained from gel-filtration chromatography as described above (Supplementary Fig. S4 and Table 3 ▶). BGLPf-M4b may exist in a dynamic monomer–dimer equilibrium. BGLPf-M3 exhibited a large mean radius with a broad monomodal size distribution (16.8 ± 19.2 nm; Supplementary Fig. S5*b*), which could be attributed to an equilibrium between dimeric and polymeric states under these conditions.

### 3.6. Thermostabilities of the mutants   

**Figure 5:**
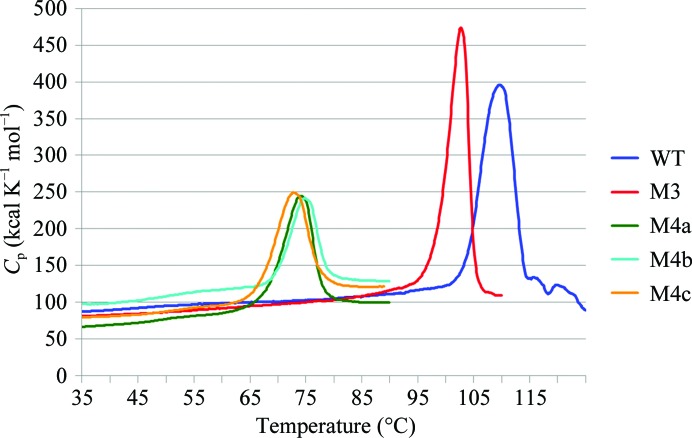
Thermal stability of BGLPf-WT (WT), BGLPf-M3 (M3), BGLPf-M4a (M4a), BGLPf-M4b (M4b) and BGLPf-M4c (M4c). The DSC results are shown as excess heat capacity, *C*_p_ (kcal K^−1^ mol^−1^), *versus* temperature (°C) profiles. The proteins (1.0 mg ml^−1^) were dissolved in 50 m*M* sodium phosphate buffer pH 7.0. The *T*_m_ temperatures of BGLPf-WT, BGLPf-M3, BGLPf-M4a, BGLPf-M4b and BGLPf-M4c were estimated as 109.5, 102.0, 74.0, 75.0 and 73.0°C, respectively.

Differential scanning calorimetry (DSC) was used to examine the thermo­stabilities of the mutants (at 1.0 mg ml^−1^ in 50 m*M* sodium phosphate buffer pH 7.0). Tetrameric BGLPf-WT had a melting temperature (*T*_m_) of about 110°C and dimeric BGLPf-M3 had a *T*_m_ value of 102°C. On the other hand, the monomeric mutants BGLPf-M4a, BGLPf-M4b and BGLPf-M4c had *T*_m_ values between 73 and 75°C (Fig. 5 ▶).

**Figure 6:**
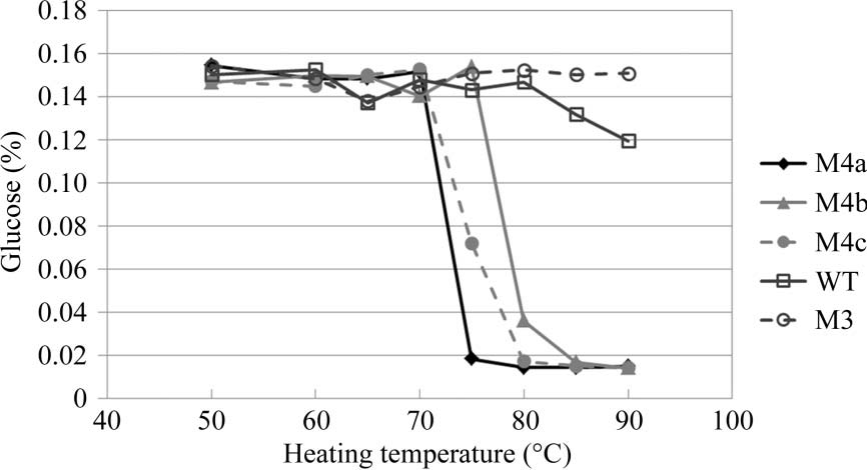
Residual activities of BGLPf-M4a (M4a), BGLPf-M4b (M4b), BGLPf-M4c (M4c), BGLPf-WT (WT) and BGLPf-M3 (M3) after heating. Purified enzymes (1.0 mg ml^−1^) were incubated for 10 min in 50 m*M* Tris–HCl buffer pH 7.2 at the given temperatures. The residual activity of each enzyme after heating was assayed under standard conditions with 10 m*M* cellobiose for 10 min at 40°C. Activity was expressed as the concentration of glucose produced (%).

We also evaluated residual activity after heating to confirm the thermostability of BGLPf-WT and its mutants. As shown in Fig. 6 ▶, BGLPf-WT and BGLPf-M3 were stable beyond 85°C, whereas BGLPf-M4a, BGLPf-M4b and BGLPf-M4c were immediately inactivated between 70 and 80°C. These data are consistent with the *T*_m_ values measured by DSC as described above, and show that the oligomerization of BGLPf provides thermostability.

## 4. Discussion   

### 4.1. A crystal of BGLPf-M3 provides high-resolution data   

Crystals of BGLPf are easily prepared and grow quickly under suitable conditions. Many kinds of precipitants from commercially available screening kits or from series of hand-made solutions were applicable for crystallization, and the crystals had an excellent appearance, with a hexagonal bipyramidal shape with a sharpened edge. However, high-resolution X-ray data have not previously been obtained from these crystals: only low-resolution data to less than 5 Å resolution have been obtained. Kaper and coworkers barely succeeded in structure determination from low-resolution data at 3.3 Å (Kaper *et al.*, 2000 ▶). Finally, Kado and coworkers succeeded in building a structural model of BGLPf at the low resolution of 2.35 Å by using a dehydration treatment (Heras & Martin, 2005 ▶; Kado *et al.*, 2011 ▶).

Knowledge of the precise structure of an enzyme is essential for understanding its enzymatic characteristics. The BGLPf crystal required a dehydration treatment (Heras & Martin, 2005 ▶) prior to X-ray diffraction analysis. Substitutive mutations based on the crystal structure were introduced into BGLPf-WT and provided important structural insights by disrupting the tetrameric structure and providing a new dimeric form of the protein: BGLPf-M3. BGLPf-M3 was crystallized in a new crystal form; it provided higher resolution X-ray diffraction data (1.70 Å) than had previously been obtained for BGLPf and showed that the tetrameric structural form of BGLPf is the cause of the low quality of the crystals. Preparation of proteins to obtain a high-quality crystal is the major bottleneck in solving protein structures, and substitutive mutations are frequently used to resolve this problem. There are two common approaches: the surface-entropy reduction method (Derewenda, 2004 ▶) and the synthetic symmetrization method (Banatao *et al.*, 2006 ▶; Laganowsky *et al.*, 2011 ▶). The latter method aims to change monomeric proteins into oligomeric proteins (Yamada *et al.*, 2007 ▶; Forse *et al.*, 2011 ▶; Laganowsky *et al.*, 2011 ▶) because oligomeric proteins generally crystallize more easily. Therefore, our result that BGLPf-M3 provides excellent crystals is unusual and is likely to be a consequence of its crystal packing. The *PISA* program identified several residues that contribute to crystal packing. Based on our available data, we present here the packing differences observed between BGLPf-WT and BGLPf-M3.

The packing of BGLPf-WT exhibits space group *P*4_3_2_1_2. *PISA* indicated six kinds of interactions (Table 4 ▶ *a*; interactions WT-5, WT-6, WT-7, WT-8, WT-9 and WT-10) contributing to crystal packing and four kinds of interactions (Table 4 ▶ *a*; interactions WT-1, WT-2, WT-3 and WT-4) contributing to the tetrameric structure. Of these interactions, three (WT-5, WT-6 and WT-7) are necessary for crystal growth and stability: WT-5 is an *A*–*B* interaction that enlarges the crystal along the *c* axis exhibiting 4_3_ helical symmetry, and WT-6 and WT-7 are *C*–*C* and *C*–*D* interactions that enlarge the crystal perpendicular to the *c* axis (Supplementary Fig. S6). The WT-5 interaction is rather stable because its contact area is large (685.1 Å^2^) and Δ^*i*^ *G* is negative. In contrast, WT-6 and WT-7 are unstable because their contact areas are rather small (282.1 and 262.8 Å^2^, respectively) and Δ^*i*^ *G* is positive. These values suggest that the crystal-packing interactions of BGLPf-WT perpendicular to the *c* axis are weak. Packing is strengthened by dehydration, since this treatment improved the quality of the diffraction data in previous experiments.

Our data indicated that the packing of BGLPf-M3 is in space group *C*2. *PISA* indicated nine kinds of interactions (Table 4 ▶ *b*; interactions M3-3, M3-4, M3-5, M3-6, M3-7, M3-8, M3-9, M3-10 and M3-11) contributing to crystal packing and two kinds of interactions (Table 4 ▶ *b*; interactions M3-1 and M3-­2) contributing to the dimeric structure. The BGLPf-M3 crystal is stabilized mainly by the M3-3, M3-4, M3-5, M3-6 and M3-7 interactions. The M3-3 and M3-4 interactions are similar to WT-5. M3-5, M3-6 and M3-7 stabilize pseudo-tetramer formation and also contribute to stable crystal packing. Since most BGLPf-M3 interactions involved in packing occur over quite large contact areas (greater than ∼500 Å^2^) and exhibit negative Δ^*i*^ *G* values, the BGLPf-M3 crystal is more stable than the BGLPf-WT crystal.

**Figure 7:**
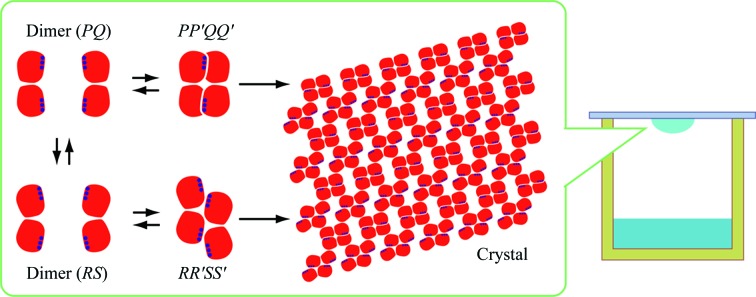
Crystallization scheme for BGLPf-M3 as inferred from its crystal packing. The BGLPf-M3 molecules form at least two distinct dimeric structures (*PQ* and *RS*) which they fluctuate between. BGLPf-M3 can also form two distinct transient pseudo-tetramers (*PP*′*QQ*′ and *RR*′*SS*′). These pseudo-tetramers are preferentially trapped and crystallize under our crystallization conditions while the dimers and tetramers are in equilibrium.

It has been reported that mutations at crystal-packing interfaces influence X-­ray diffraction quality (Mizutani *et al.*, 2008 ▶). The effect of crystal packing and intermolecular interactions can significantly influence the quality of the crystal. The diffraction data from BGLPf-M3 at 1.70 Å resolution indicate well ordered lattice molecules owing to stable intermolecular interactions in the crystal. BGLPf-M3 seems to form two different types of dimeric structure (*P*–*Q* and *R*–*S*) as identified by structural analysis (Figs. 4 ▶ *b* and 4 ▶ *c*). The interface between the two monomer forms appears to be flexible, like a hinge, and the flexible dimer structure seems to contribute to a stable, well packed crystal structure that is different from the stable tetrameric structure of BGLPf-WT. Our crystal structure suggests the following crystallization process for BGLPf-M3. Initially, most BGLPf-M3 molecules exist in the dimeric state in solution and can fluctuate between two dimeric states because of the transient interactions mediated by Arg381, Tyr382, Arg448 and Glu449. The two dimeric structures co-exist in equilibrium and both occasionally form two types of tetrameric states which are trapped during the crystallization process (Fig. 7 ▶). BGLPf-M3 provides high-resolution X-ray diffraction data and thus might be useful for the structural analysis of BGLs bound to ligands such as substrate analogues and inhibitors.

### 4.2. Design of monomeric BGLPf mutants   

Analysis of the crystal structure and other characteristics indicated that BGLPf-M3 adopts alternate dimeric forms and that mutation of Arg448, Glu449 and Glu459 was necessary to favour the monomeric state. In particular, one dimeric state of BGLPf-M3 is tethered by Arg381–Tyr382 and the other state is tethered by Arg448–Glu449. Formation of Arg381–Tyr382 prevents the formation of Arg448–Glu449 and *vice versa*. Thus, the oligomeric structure of M3 can be disrupted by only one additional mutation. The introduction of single substitutive mutations such as R448E or E459G into BGLPf-M3 was sufficient to eliminate the dimeric state and promote the monomeric state.

### 4.3. Oligomerization of BGLPf contributes to its thermostability   

Monomeric BGLPfs with activities comparable to that of WT were obtained, demonstrating that the mutations used to convert the tetramer into the monomer did not cause any loss of enzymatic activity. However, the thermostability of the protein suffered. Nonetheless, the *T*_m_ values of the monomeric BGLPfs (approximately 75°C) are higher than those of mesophilic BGLs from the bacterium *Clostridium cellulovorans*, the fungus *T. reesei* and the termite *Neotermes koshunensis* (Jeng *et al.*, 2011 ▶). Several factors responsible for the thermostability of proteins have been proposed as increasing numbers of crystal structures of proteins from thermophilic organisms have been reported. Examples include oligomerization driven by several subunits, the accumulation of ion pairs and hydrogen bonds on the protein surface, increasing hydrophobicity and packing density in the protein core, and an entropic effect caused by shortened surface loops or the introduction of proline residues into loops. We modified one of these factors: the oligomeric state. Below, we discuss the observation that the substitutive mutations exerted negative effects on thermostability.

Tetrameric BGLPf-WT derived from *P. furiosus*, an anaerobic bacterium that grows between 70 and 103°C, has a *T*_m_ of about 110 °C. Consequently, BGLPf-WT does not completely denature during boiling in denaturation agents. Dimeric BGLPf-M3 has a *T*_m_ value of about 100°C; this decrease in *T*_m_ is apparently caused by dissociation of the tetrameric state owing to the three substitutive mutations. A striking difference between BGLPf-M3 and monomeric BGLPfs (including the four substitutive mutations) is that the *T*_m_ of the monomers is between 73 and 75 °C.

The difference in *T*_m_ values between BGLPf-M3 and the monomeric mutants can be explained as follows. Inter-subunit contacts in BGLPf-M3 involve hydrophobic interactions mediated by Pro384, Pro442, Leu445 and Val446 and hydrophilic interactions mediated by Arg381, Tyr382, Arg448 and Glu449, as discussed above. The hydrophobic interactions in the centre of the dimer interface are retained in both the *PQ* and *RS* dimers, in contrast to the hydrophilic interactions in the solvent-exposed region, which are variable. Thus, dimer formation is not conserved amongst mesophilic BGLs (Jeng *et al.*, 2011 ▶; PDB entries 3ahx, 3ahy, 3ahz and 3ai0): in these proteins the subunits are not tethered by hydrophobic residues which contribute to thermostability. The lack of such hydrophobic contacts would result in an altered *T*_m_. While we previously predicted that oligomerization of BGLPf would increase its thermostability (Kado *et al.*, 2011 ▶), this was verified by the experiments described above. Crystal structure determinations of monomeric BGLPs are under way and will allow us to design more thermostable monomers.

## Supplementary Material

PDB reference: BGLPf-M3, 3wdp

Supporting Information.. DOI: 10.1107/S1399004713032276/tz5040sup1.pdf

## Figures and Tables

**(a):** *A*–*C* interactions in BGLPf-WT.

	Hydrogen bonds		
Interaction	Subunit *A*	Distance (Å)	Subunit *C*
AC-1	Arg381 NH1	3.01	Tyr382 OH
AC-2	Tyr382 OH	2.97	Arg381 NH1
AC-3	Leu440 N	2.98	Glu459 O
AC-4	Glu459 O	2.86	Leu440 N
AC-5	Gly332 O	2.86	Arg471 NH2
AC-6	Arg471 NH2	3.15	Gly332 O

**(b):** *P*–*Q* interactions in BGLPf-M3.

	Hydrogen bonds		
Interaction	Subunit *P*	Distance (Å)	Subunit *Q*
PQ-1	Arg381 NH1	2.92	Tyr382 OH
PQ-2	Tyr382 OH	2.89	Arg381 NH1
PQ-3	Leu440 N	2.89	Glu459 O
PQ-4	Glu459 O	2.89	Leu440 N

**(c):** *R*–*S* interactions in BGLPf-M3.

	Hydrogen bonds or salt bridges		
Interaction	Subunit *R*	Distance (Å)	Subunit *S*
RS-1	Arg448 NH2	2.99	Glu449 OE1
RS-2	Glu449 OE1	2.97	Arg448 NH2
RS-3	Leu440 N	2.86	Glu459 O
RS-4	Glu459 O	2.89	Leu440 N

**Table 2:** Summary of statistics and refinement of the BGLPf-M3 crystallographic data Values in parentheses are for the highest resolution shell.

PDB entry	3wdp
X-ray source	BL44XU, SPring-8
Wavelength (Å)	0.90
Space group	*C*2
Unit-cell parameters (Å, °)	*a* = 122.15, *b* = 161.15, *c* = 183.82, α = 90.00, β = 108.38, γ = 90.00
Resolution range (Å)	50.00–1.70 (1.76–1.70)
Total No. of reflections	1214951
No. of unique reflections	367711
Completeness (%)	99.3 (99.1)
Mean *I*/σ(*I*)	20.0 (3.9)
*R*_merge_ †	0.086 (0.361)
Refinement statistics	
Resolution range (Å)	50.00–1.70 (1.76–1.70)
*R* factors‡ (*R*_free_/*R*_work_)	0.233/0.212 (0.281/0.258)

† *R*_merge_ = 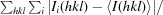 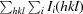, where 〈*I*(*hkl*)〉 is the mean intensity of all reflections equivalent to reflection *hkl*.

‡ *R*_work_ and *R*_free_ = 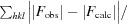 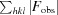, where a randomly selected 5% of the data were used to calculate *R*_free_.

**Table 3:** Molecular size and putative oligomeric state of wild-type and mutant BGLPf The theoretical molecular mass of monomeric BGLPf estimated from the amino-acid sequence is 55 kDa. Therefore, the gel filtration suggests that BGLPf-WT is tetrameric, BGLPf-M3 is dimeric and BGLPf-M4a and BGLPf-M4c are monomeric. These data are also supported by the DLS measurements except for BGLPf-M3, which does not show a dimeric size.

	BGLPf-WT	BGLPf-M3	BGLPf-M4a	BGLPf-M4b	BGLPf-M4c
Molecular mass (gel filtration) (kDa)	236	136	71	96	50
Radius (DLS) (nm)	5.7 ± 1.1	16.8 ± 19.2	4.0 ± 1.7	4.1 ± 1.6	3.2 ± 1.0
Putative oligomeric state	Tetramer	Dimer	Monomer	Dimer or monomer	Monomer

**(a):** BGLPf-WT.

	Structure 1		Structure 2				
Interaction	Subunit	Surface (Å^2^)	Subunit	Symmetry operation	Surface (Å^2^)	Interface area (Å^2^)	Δ^*i*^ *G* (kcal mol^−1^)
WT-1	*C*	18288	*A*	*x*, *y*, *z*	18262	1197.4	−10.7
WT-2	*D*	18475	*B*	*x*, *y*, *z*	18358	1196.6	−11.7
WT-3	*B*	18358	*A*	*x*, *y*, *z*	18262	943.1	−4.0
WT-4	*D*	18457	*C*	*x*, *y*, *z*	18288	937.1	−7.0
WT-5	*A*	18262	*B*	−*y* + 1/2, *x* − 1/2, *z* − 1/4	18358	685.1	−3.6
WT-6	*C*	18288	*C*	*y* + 1, *x* − 1, −*z*	18288	282.1	3.3
WT-7	*D*	18457	*C*	*y* + 1, *x* − 1, −*z*	18288	262.8	0.9
WT-8	*D*	18457	*A*	*y*, *x* − 1, −*z*	18262	250.4	2.2
WT-9	*D*	18457	*B*	*y*, *x* − 1, −*z*	18358	116.8	0.0
WT-10	*C*	18288	*A*	−*x* + 1/2, *y* − 1/2, −*z* − 1/4	18262	27.2	0.9

**(b):** BGLPf-M3.

	Structure 1		Structure 2				
Interaction	Subunit	Surface (Å^2^)	Subunit	Symmetry operation	Surface (Å^2^)	Interface area (Å^2^)	Δ^*i*^ *G* (kcal mol^−1^)
M3-1	*Q*	17961	*P*	*x*, *y*, *z*	18007	1124.8	−12.9
M3-2	*S*	17996	*R*	*x*, *y*, *z*	18007	903.2	−10.6
M3-3	*P*	18007	*S*	*x* − 1/2, *y* + 1/2, *z*	17996	765.8	−0.2
M3-4	*R*	18007	*Q*	*x*, *y*, *z*	17961	757.3	0.6
M3-5	*S*	17996	*R*	−*x* + 1, *y*, −*z* + 1	18007	821.7	−10.8
M3-6	*Q*	17961	*Q*	−*x*, *y*, −*z*	17961	507.9	−7.9
M3-7	*P*	18007	*P*	−*x*, *y*, −*z*	18007	498.3	−7.8
M3-8	*Q*	17961	*P*	*x* − 1/2, *y* − 1/2, *z*	18007	427.0	−1.1
M3-9	*S*	17996	*S*	−*x* + 1, *y*, −*z* + 1	17996	216.0	2.2
M3-10	*Q*	17961	*P*	−*x*, *y*, −*z*	18007	68.3	1.5
M3-11	*R*	18007	*R*	−*x* + 1, *y*, −*z* + 1	18007	26.3	−0.8

## Footnotes

1 Supporting information has been deposited in the IUCr electronic archive (Reference: TZ5040).
