# Supplementary material for: Structural analysis of β-glucosidase mutants derived from a hyperthermophilic tetrameric structure
Source: Acta Crystallogr D Biol Crystallogr. 2014 Feb 27;70(Pt 3):877–88. doi: 10.1107/S1399004713032276 (PMC3949513; doi:10.1107/S1399004713032276)
Supplement: Supplementary file 1 [file d-70-00877-sup1.pdf]

Supporting Information

Structural analysis of β-glucosidase mutants derived from a hyperthermophilic tetrameric structure

Makoto Nakabayashi, Misumi Kataoka, Yumiko Mishima, Yuka Maeno and Kazuhiko Ishikawa

Figure S1
Tetrameric structure of the BGLPf-WT

Figure S2
Stereo diagram of inter-subunit interactions between A-B of the BGLPf-WT

Figure S3
A *cis*-type peptide bond was observed between Trp410 and Ser411 in each subunit.

Figure S4
Gel-filtration profiles of the BGLPf-WT and the mutants

Figure S5
DLS size distributions of the BGLPf-WT, M3, M4a, M4b and M4c

Figure S6
Crystal-packing of the BGLPf-WT

Table S1
Wild type and substitutive mutants of BGLPf discussed in this paper

Table S2
Amino acid residues involved in an inter-subunit interaction between A-B of the BGLPf-WT

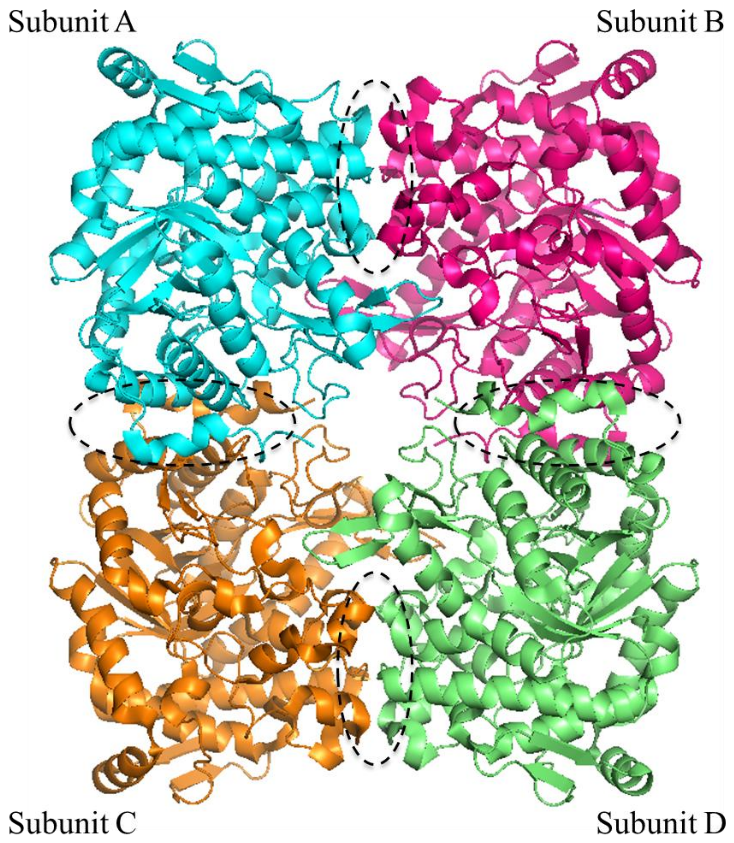

Figure S1
Tetrameric structure of the BGLPf-WT

The subunits A, B, C and D are shown to be separated by colors, cyan, pink, orange and lime, respectively. Two types of inter-subunit interactions are shown by dashed ellipses. Interaction of the A-B is almost identical to that of the C-D. Interaction of the A-C is almost identical to that of the B-D.

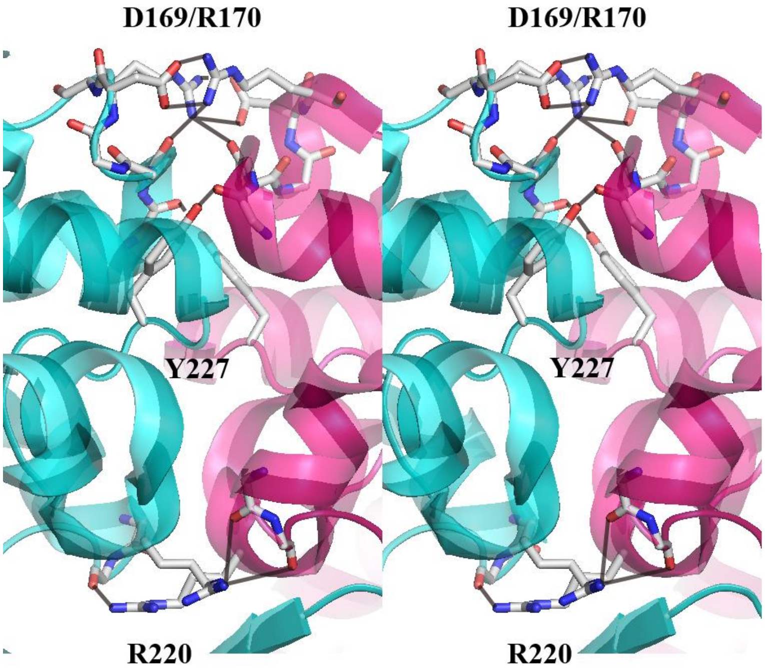

Figure S2

Stereo diagram of inter-subunit interactions between the subunits A-B of the BGLPf-WT

The subunits A and B are shown as the cyan and pink ribbon models, respectively. Side chains of Asp169, Arg170, Tyr227 and Arg220 are shown as stick models to be separated by atom colors (white carbon, blue nitrogen and red oxygen). Several atoms of main chain are also shown by the stick models. Putative interactions are shown by black solid lines.

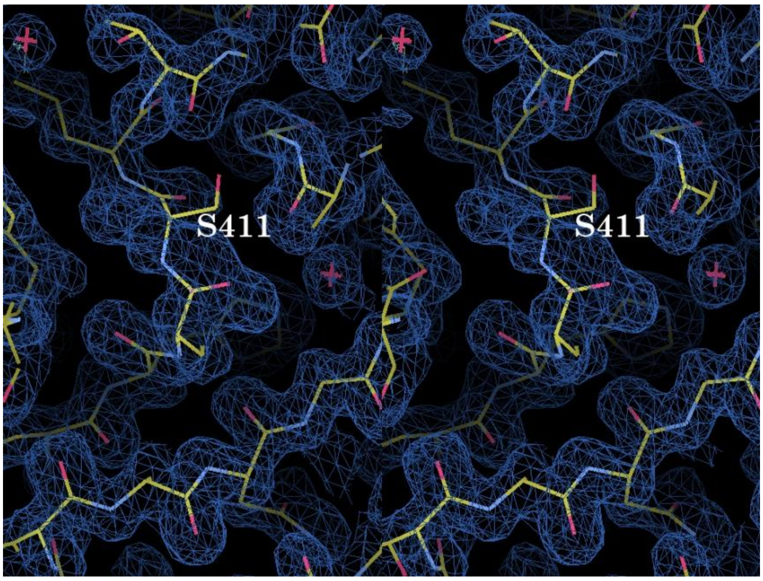

Figure S3

A *cis*-type peptide bond was observed between Trp410 and Ser411 in each subunit.

A part of the subunit P for example is shown by the stereo diagram. The structure is colored by atom type (C, yellow; O, red; N, blue). $2F_{obs}$-F_{calc} map contoured at 2.0σ is drawn by blue. The 411th serine residue is shown by the label ‘S411’. Model coordinates with *trans*-type peptide bond was applied as a test between Trp410 and Ser411.

The high resolution data rather than the previous one clearly indicated that the *trans*-type peptide bond was not correct.

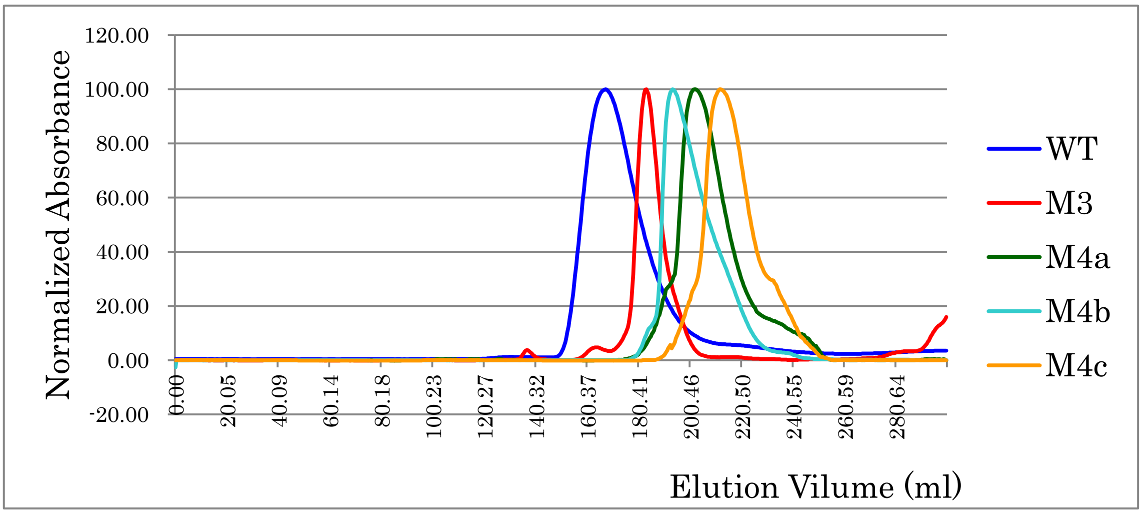

Figure S4

Gel-filtration profiles of the BGLPf-WT and the mutants

Elution profiles of the BGLPf-WT, M3, M4a, M4b and M4c are shown by blue, red, green, cyan and orange lines, respectively. Elution volumes of the WT, M3, M4a, M4b and M4c obtained from the profiles are 167.62, 183.40, 202.16, 193.63 and 211.97 ml, respectively. Molecular masses of the WT, M3, M4a, M4b and M4c estimated from the elution volumes are 236 kDa, 136 kDa, 71 kDa, 96 kDa and 50 kDa, respectively.

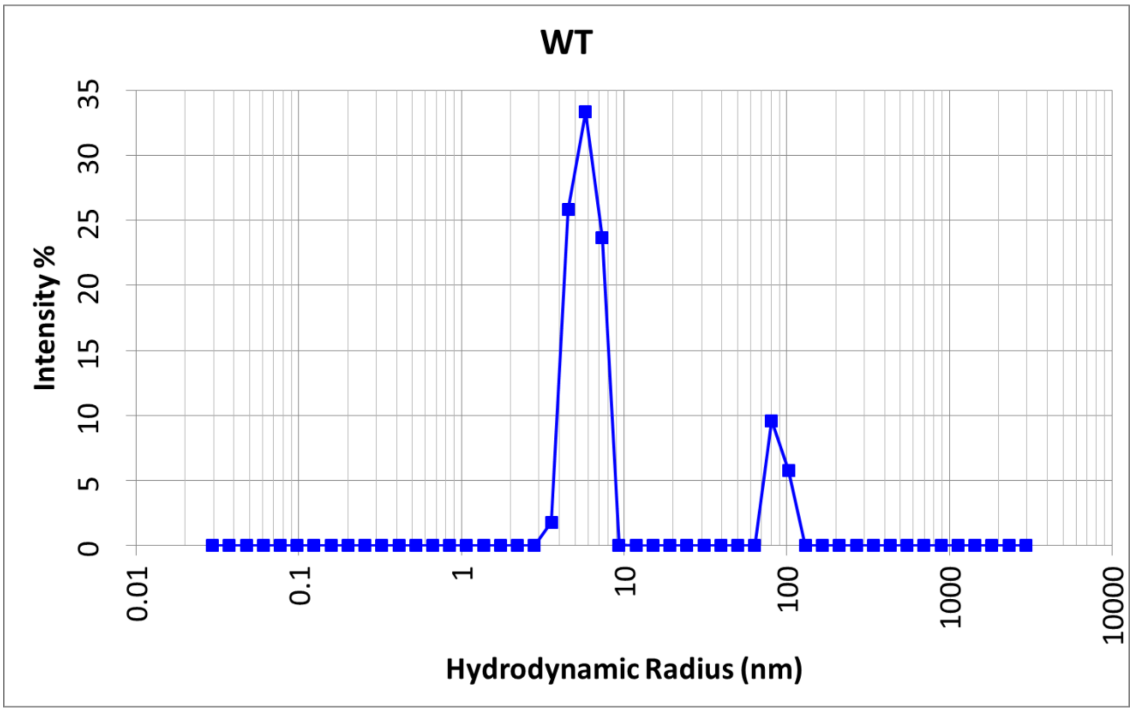

Figure S5(*a*)

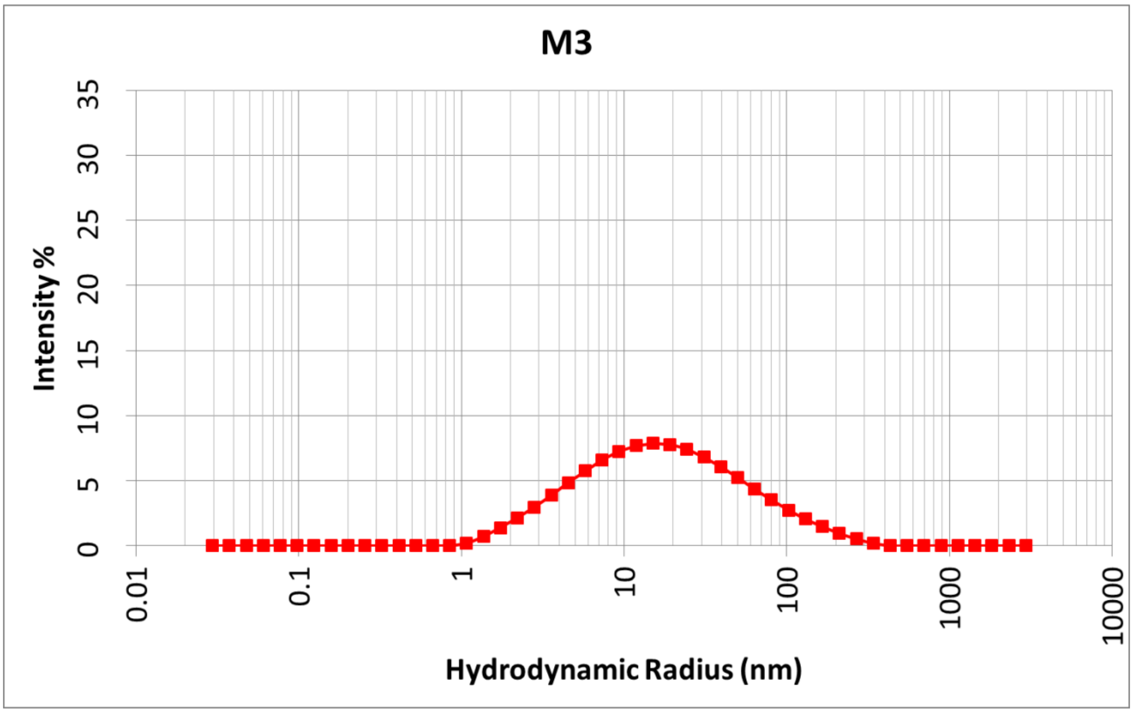

Figure S5(*b*)

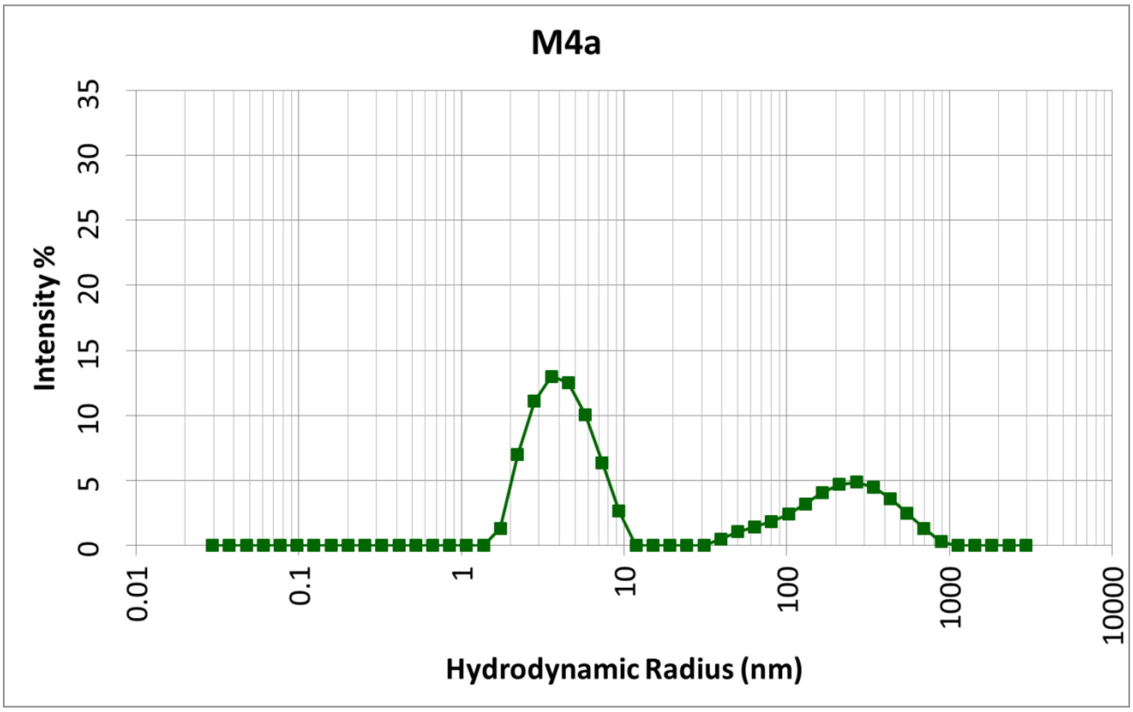

Figure S5(*c*)

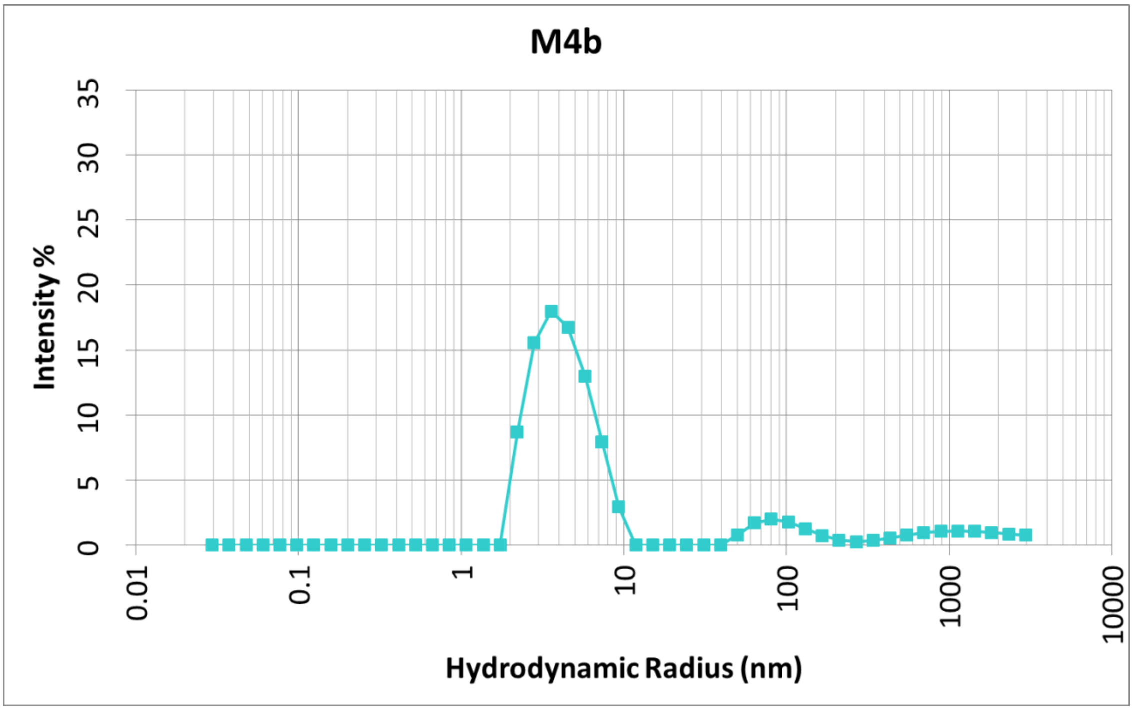

Figure S5(*d*)

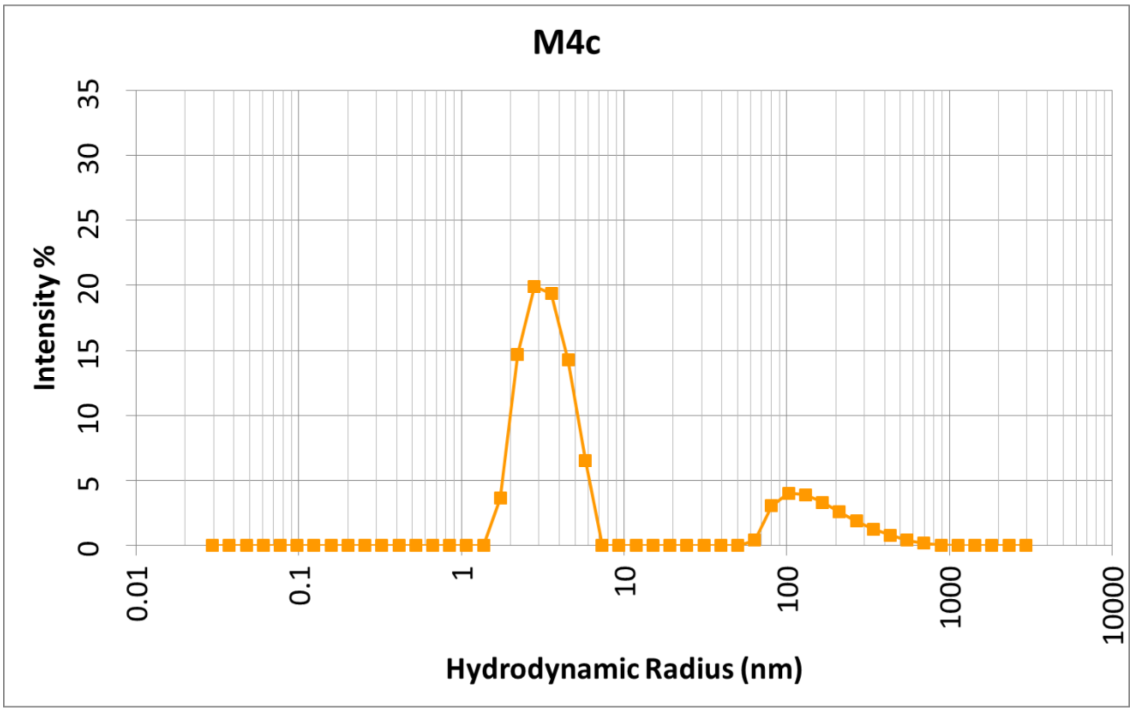

Figure S5(*e*)

Figure S5

DLS size distributions of the BGLPf-WT (S5A), M3 (S5B), M4a (S5C), M4b (S5D) and M4c (S5E)

The results of the WT, M4a, M4b and M4c showed multimodal shaped distributions in contrast to the M3 whose result showed a broad mono-modal one. The hydrodynamic radii of the WT, M3, M4a, M4b and M4c are estimated to be 5.7 ± 1.1 nm, 16.8 ± 19.2 nm, 4.0 ± 1.7 nm, 4.1 ± 1.6 nm and 3.2 ± 1.0 nm, respectively.

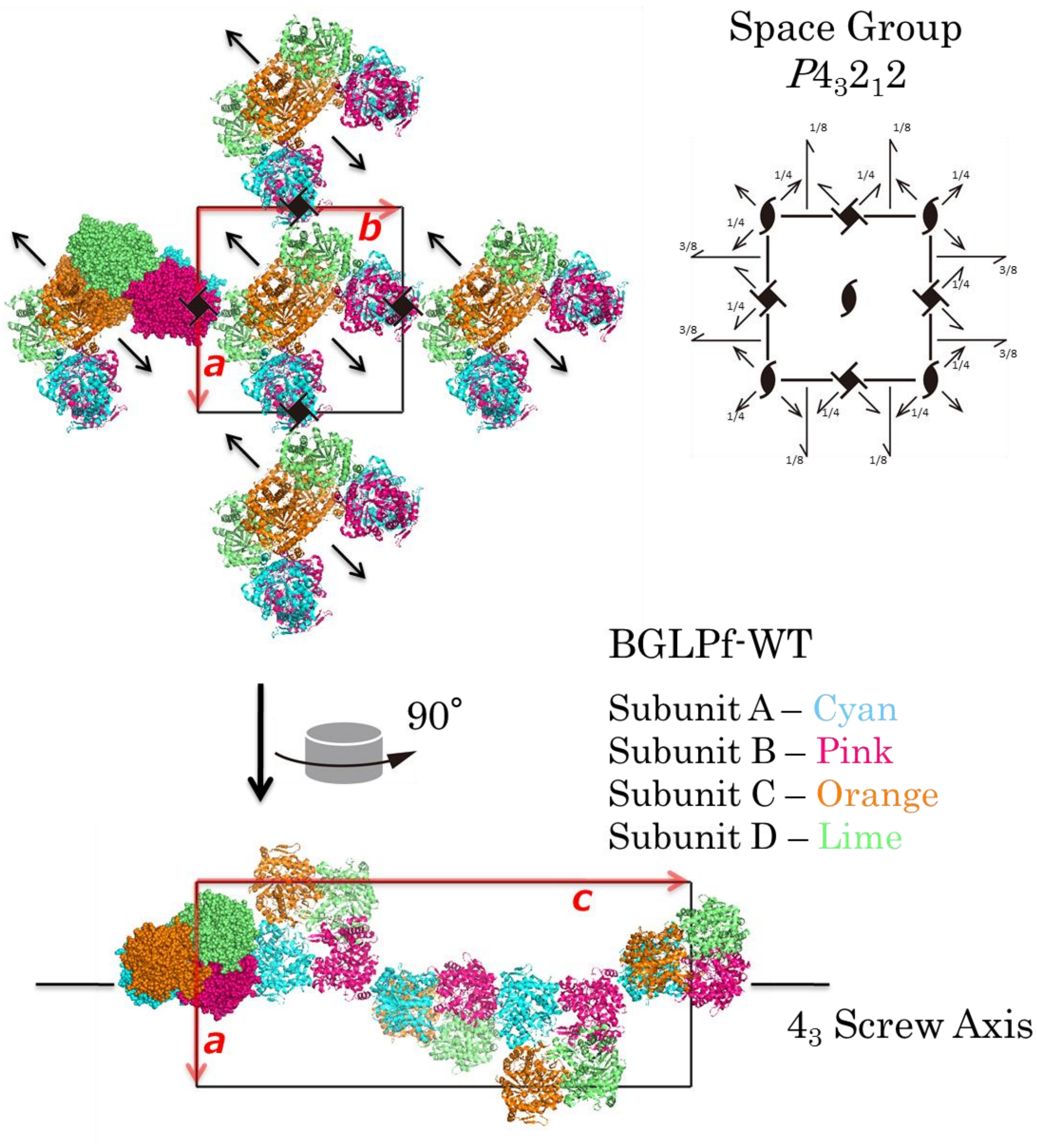

Figure S6
Crystal-packing of the BGLPf-WT

Unit cell is shown as black square or rectangle. Lattice vectors, ***a***, ***b*** and ***c*** are shown as red arrows. One tetramer existing in the asymmetric unit is shown as spacefil model with colors of cyan (subunit A), pink (subunit B), orange (subunit C) and lime (subunit D). The other molecules related by the crystallographic symmetry are shown as ribbon model (upper left and under diagrams).

An A-B interaction, one of inter-molecular interactions enlarges the crystal along *c*-axis exhibiting the 4_3 helical symmetry (under diagram). The A-B interaction corresponds to the interaction ID

WT-5 in the Table 4A. Other inter-molecular interactions are C-C and C-D, which enlarge the crystal perpendicular to the *c*-axis (upper left diagram). The C-C and C-D interactions correspond to the interaction IDs WT-6 and WT-7 in the Table 4A, respectively.

Expediential Name	Substitutive Mutations
WT	none
M3	R170A & R220A & Y227F
M4a	R170A & R220A & Y227F & R448E
M4b	R170A & R220A & Y227F & E449R
M4c	R170A & R220A & Y227F & E459G

Table S1
Wild type and substitutive mutants of BGLPf discussed in this paper

Expediential names and the comrresponding mutations are shown in left and right columns, respectively.

	Hydrogen Bonds or Salt Bridges		
Interaction ID	Subunit A	Distance [Å]	Subunit B
AB-1	ARG 170 [NH1]	3.01	LEU 166 [O]
AB-2	LEU 166 [O]	3.12	ARG 170 [NH1]
AB-3	ARG 170 [NH2]	2.59	ASP 169 [OD1]
AB-4	ASP 169 [OD2]	2.67	ARG 170 [NH2]
AB-5	GLY 44 [O]	2.92	ARG 220 [NH2]
AB-6	TYR 227 [OH]	2.62	LYS 165 [O]
AB-7	LYS 165 [O]	2.76	TYR 227 [OH]
AB-8	PHE 230 [N]	2.98	GLU 39 [OE1]
AB-9	GLU 39 [OE1]	3.06	PHE 230 [N]

Table S2
Amino acid residues involved in an inter-subunit interaction between the subunits A-B of the BGLPf-WT.

Arg170, Arg220 and Tyr227 are highlighted by yellow backgrounds. Thus, side chains of the Arg170, Arg220 and Tyr227 mainly contribute to the A-B interaction.
